# Transcranial magnetic stimulation in migraine prophylaxis

**Published:** 2018

**Authors:** P Leahu, A Matei, S Groppa

**Affiliations:** * “N. Testemitanu” State Medical and Pharmaceutical University; ** Institute of Emergency Medicine

**Keywords:** Transcranial, magnetic, stimulation, TMS, migraine, prophylaxis

## Abstract

Transcranial Magnetic Stimulation (TMS) is a non-invasive brain stimulation method used worldwide to make causality-based inferences about brain-behavior interactions, assess cortical reactivity, and map functionally relevant brain regions inducing a controlled current pulse in a specific cortical area. Clinical applications of TMS have shown promising results in the treatment of a vast number of psychiatric and neurological conditions such as headache disorders - migraine being one of the most encountered. In patients with migraine, the pharmacologic therapy is divided in urgent/ abortive treatment of the attack and prophylactic one. As first-line drugs simple analgesics and non-steroidal inflammatory are preferred. Nevertheless, many individuals continue to have attacks refractory to various prophylactic and/or abortive therapies, while others are at high risk of developing medication overuse headache. Among non-pharmacologic therapies TMS has been broadly studied as a preventive migraine treatment with good outcome results.

**Abbreviations:** DLPFC - Dorsolateral prefrontal cortex, FDA – United States Food and Drug Administration, HF-TMS – High frequency transcranial magnetic stimulation, TMS – Transcranial magnetic stimulation, rTMS – Repetitive transcranial magnetic stimulation

Single-pulse transcranial magnetic stimulation (TMS) was introduced for the first time in 1985, as a method of noninvasive and painless stimulation of the human cortex [**1**][**2**]. This technique opened up new ways of studying the functionality, morphology, and connectivity of various cortical regions, especially the motor cortex [**3**]. In 1990, technological advances introduced generators capable of producing rapid, repetitive pulses of magnetic stimulation at frequencies of up to 30 Hz known as repetitive TMS (rTMS) or high-frequency TMS (HF-TMS) [**4**]. Currently, rTMS is considered a useful tool in the treatment of several conditions originating in the cerebral cortex including pain, dystonia, epilepsy, headaches, Parkinson’s disease, stroke, and tinnitus. Although the exact mechanisms through which rTMS works are not completely known at the moment, it is considered to be multifactorial. The applied electric charges induced by the magnetic field cause various neurochemical changes such as increased dopamine levels in the hippocampus, reduction in Raclopride C11 binding in the caudate nucleus, fluctuations in glutamate/glutamine levels at the site of rTMS stimulation and increased plasma β-endorphin levels [**5**][**6**].

Considering the pathophysiology of migraine and the fact that the incidence of migraine is gradually rising and becoming one of the most common nervous system diseases in the world [**7**], very often being associated with affective disorders such as anxiety and depression, there has been an increased interest in using rTMS in the management of these patients applying different TMS paradigms and protocols [**8**][**9**]. There are several studies that confirmed the fact that people with migraine (“migraineurs”) had 2.2 to 4.0 times increased fold of developing depression than non-migraineurs [**10**]. Regarding the effects of TMS in migraine, previous studies have also suggested that blood flow [**11**] and metabolic changes [**12**] at the stimulation site, brain-derived neurotrophic factor upregulation, improvements in synaptic plasticity [**13**] and changes in the activity of the neural circuitry of the dorsolateral prefrontal (DLPFC) - cingulate cortex, including both the DLPFC and the anterior cingulate cortex [**14**], are involved. There is also research data that confirms a positive effect of HF-TMS on cognitive tasks in healthy adults [**15**] suggesting that TMS can not only recruit more neural resources by inducing an electrophysiological excitatory effect but can also enhance the efficiency of resource deployment during multiple stages of cognitive control processing. In a position statement for the neuromodulation of chronic headaches, the European Headache Federation states that the application of noninvasive rTMS in chronic headaches is not yet evidence-based, given the limited amount of randomized controlled data and can’t be used on a regular basis for all migraine patients [**16**]. Although there are multiple positive and promising conducted studies regarding the use of TMS in migraine prophylaxis and FDA approved devices indicated for the acute treatment of pain associated with migraine headache with aura [**17**], evidence on the efficacy of TMS for the treatment of migraine is still limited in quantity, demanding randomized controlled trials to analyze safety of long-term or frequent use of TMS. Therefore, TMS should only be used with special arrangements for clinical governance, consent and audit or research until proven otherwise [**18**].

## Conflict of Interest

The authors declare that there is no conflict of interest.
